# Supplementary material for: Discrimination of Head and Neck Squamous Cell Carcinoma Patients and Healthy Adults by 10-Color Flow Cytometry: Development of a Score Based on Leukocyte Subsets
Source: Cancers (Basel). 2019 Jun 12;11(6):814. doi: 10.3390/cancers11060814 (PMC6628584; doi:10.3390/cancers11060814)
Supplement: Supplementary file 1 [file cancers-11-00814-s001.pdf]

Figure S1. **Gating strategy for the 10-color 13-antibody panel OMIP-023** using a sample of one subject. EDTA-anticoagulated whole blood was pre-lysed to remove $CD45^{-}$ erythrocytes and to enrich $CD45^{+}$ leukocytes. The stained peripheral blood sample was prepared for analysis by excluding air bubbles (time *versus* sideward scatter, plot 1) followed by gating on single-cell events (exclusion of non-single events outside the gate) in plot 2 (time of flight forward scatter *versus* forward scatter) before gating in plot 3 ($CD45^{+}$ leukocytes) on the three major subsets, granulocytes (A, SSC^{high}), monocytes (B, SSC^{med}) and lymphocytes (C, SSC^{low}) for further analyses (see Figure S2; plot 3 shown here corresponds to plot 1 in Figure S2). Further details can be found in *Methods** and in [15] and [16].

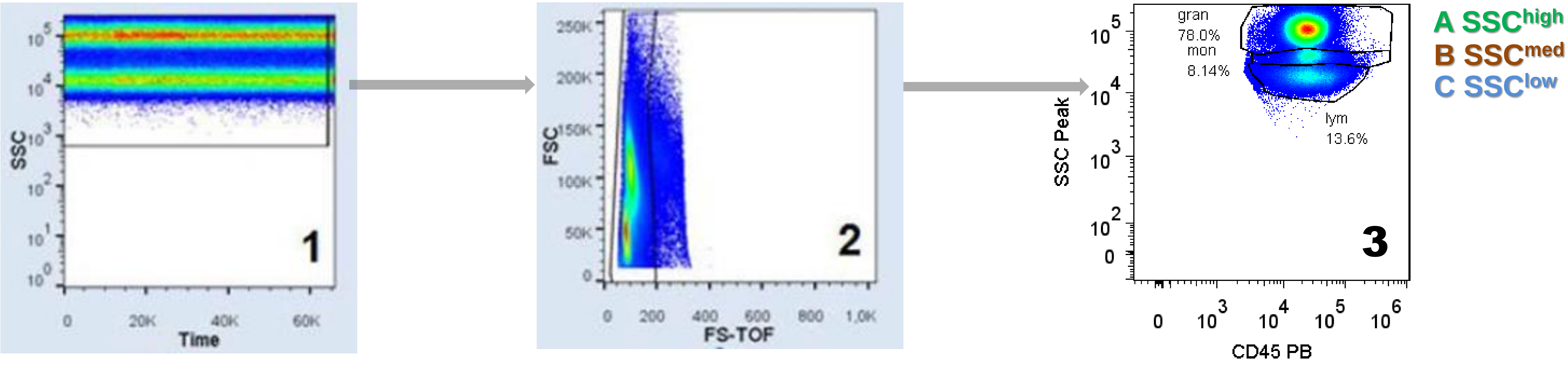

15. Bocsi, J.; Melzer, S.; Dähnert, I.; Tárnok, A. OMIP-023: 10-color, 13 antibody panel for in-depth phenotyping of human peripheral blood leukocytes. Cytometry A 2014, 85, 781–784, doi: 10.1002/cyto.a.22505.

16. Melzer, S.; Zachariae, S.; Bocsi, J.; Engel, C.; Löffler, M.; Tárnok, A. Reference intervals for leukocyte subsets in adults: Results from a population-based study using 10-color flow cytometry. Cytometry B Clin Cytom 2015, 88, 270–281, doi: 10.1002/cyto.b.21234.

Figure S2. **Gating strategy for the 10-color 13-antibody panel OMIP-023** using a sample of one subject. After excluding air bubbles and non-single events (plots 1 and 2 in Figure S1), CD45$^+$ leukocytes were gated according to SSC height to analyze granulocytes SSC^{high}; plot 2), monocytes (SSC^{med}; plots 3 to 5), and lymphocytes (SSC^{low}; plots 6 to 11). Further details can be found in *Methods** and in [15] and [16].

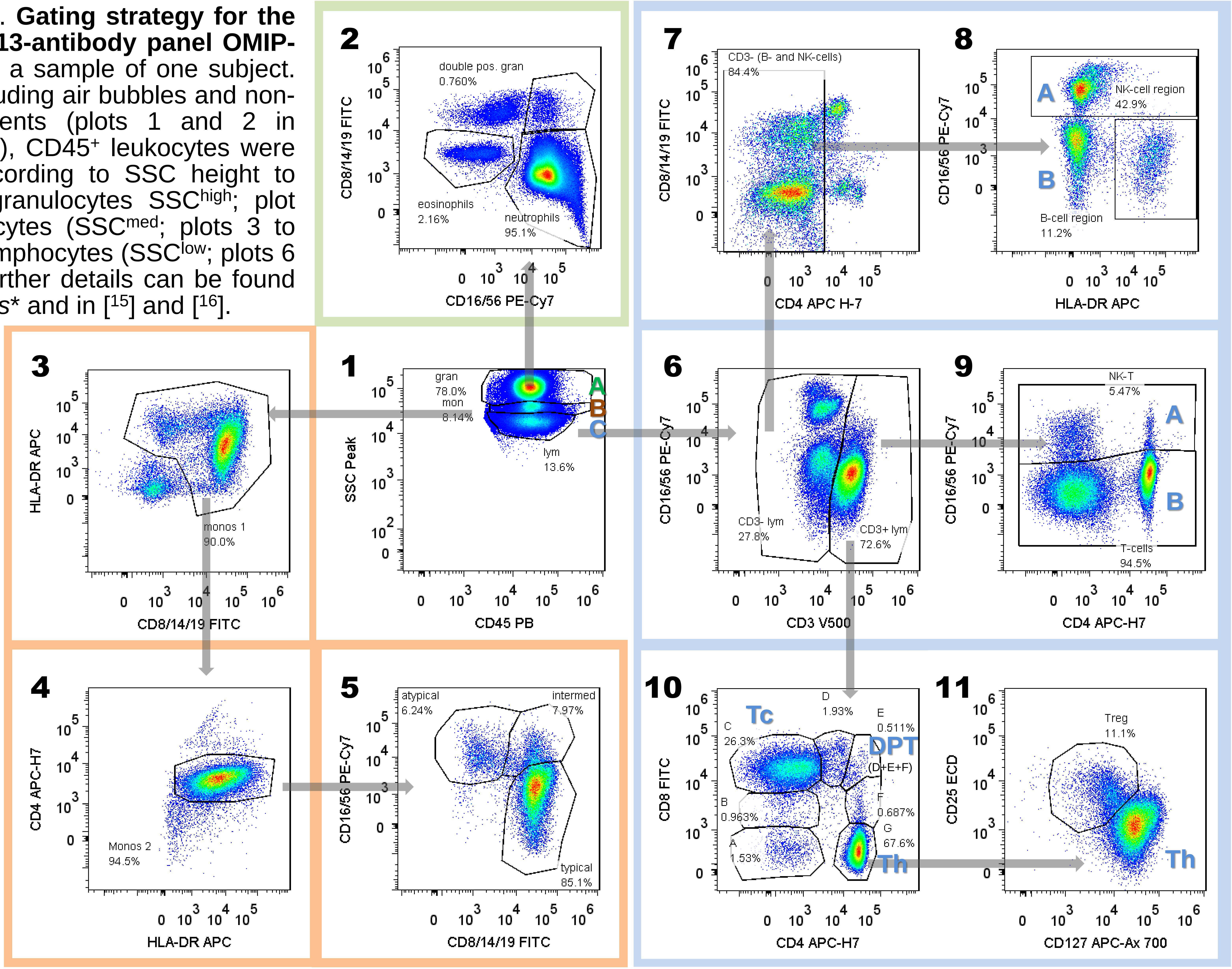

* According to SSC height, gates of granulocytes (plot 1 A: CD45$^+$SSC^{high}), monocytes (B: CD45$^+$SSC^{med}) and lymphocytes (C: CD45$^+$SSC^{low}) were discriminated and further subdivided. Neutrophils (plot 2: CD16$^+$) and eosinophils (plot 2: CD16$^-$) in granulocytes were discriminated. After excluding CD14$^-$HLA-DR$^-$ events (plot 3) and CD4$^-$ events (plot 4) from monocyte analysis, classical (typical) monocytes (plot 5: CD14^{++}CD16$^+$) and nonclassical monocytes (plot 5: atypical [CD14^{dim}CD16^{++}] and intermediate [CD14$^+$CD16^{++}]) were discriminated as well. Lymphocytes (plot 6) were gated into CD3$^-$ (left; after exclusion of CD4$^+$ events [plot 7] further analyzed in plot 8A: CD16/56$^+$ NK cells; plot 8B: CD16/56$^-$ B-lymphocytes) and CD3$^+$ events (right; further analyzed in 9A: CD3$^+$CD16/56$^+$ NKT cells and 9B: CD3$^+$CD16/56$^-$ T-lymphocytes). Three T-lymphocyte subsets were differentiated (plot 10): CD8 ^{high} cytotoxic T cells (Tc), CD4$^+$ T-helper cells (Th) and CD4$^+$CD8$^+$ double positive T-cells (DPT). The gated T-helper cells were also used to identify CD25$^+$ regulatory T-cells (Treg: anti-CD127 *versus* anti-CD25, plot 11). Further details in [15] and [16].
